# A Systematic Review of the Associations of Adult Sexual Abuse in Women with Cardiovascular Diseases and Selected Risk Factors

**DOI:** 10.5334/gh.760

**Published:** 2020-09-24

**Authors:** Nasheeta Peer, Naeemah Abrahams, Andre-Pascal Kengne

**Affiliations:** 1 Non-communicable Diseases Research Unit, South African Medical Research Council, ZA; 2 Department of Medicine, University of Cape Town, ZA; 3 Gender and Health Research Unit, South African Medical Research Council, ZA

**Keywords:** adult sexual abuse, heart attack, stroke, hypertension, diabetes, lipids, cholesterol, obesity, cardiovascular disease, cardiometabolic

## Abstract

**Aim:**

To systematically assess the association between adult sexual abuse (ASA) and cardiovascular diseases (CVDs) of heart attack and stroke, and their risk factors of hypertension, diabetes, dyslipidaemia and obesity.

**Methods:**

Two authors conducted a PubMed, Scopus and Web of Science review of the literature published prior to 1 October 2019. Relevant English language studies irrespective of study design and data collection techniques were included. Included articles needed to have examined ASA per se independent of physical or psychological/emotional abuse, and childhood sexual abuse.

**Results:**

Of the 3260 articles identified, nine were selected from 97 full-text articles assessed for eligibility (n = 855 206 women). Six studies were cross-sectional in design while three articles were from longitudinal studies; all emanated from the United States. One study only (n = 867) performed clinical assessments (heights and weights) to determine the relevant outcome (obesity), four presented self-reported outcomes (heart attack, stroke, hypertension, diabetes, obesity), two reviewed clinical records and two did not provide details on outcome assessments (obesity). Sexual abuse by an intimate partner was examined in five articles, three assessed military sexual trauma and a single study evaluated any perpetrator. A meta-analysis could not be conducted because of the heterogeneity across studies. The available evidence was insufficient to quantify the relationship, if any, between ASA and CVDs and their risk factors.

**Conclusions:**

Currently, no longitudinal studies of ASA in general populations have objectively investigated the development of CVDs and their risk factors using clinical and biochemical measurements. In view of the high CVD burden, and the frequency of ASA, it is important to fully understand the relationship between the two.

## Introduction

Over the past few decades, increasing evidence has emerged on the influence of psychosocial stress on the development of cardiovascular diseases (CVDs). Multiple studies, using different designs and approaches, have reported a relationship between psychosocial stress and CVD such as acute myocardial infarction and stroke [1,2,3], and its risk factors [1,4,5] of hypertension [5,6,7,8,9,10,11,12,13], diabetes [14] and obesity [15,16,17,18,19]. Numerous psychosocial stressors have been assessed, from external stressors such as work stress, adverse life events and financial problems, to possible reactions to stress such as depression, anxiety, psychological distress and sleeping difficulties [1,20].

A key psychosocial stressor that many women globally have been exposed to is sexual abuse which may include rape, to be defined as any form of unwanted or non-consensual sexual contact obtained by physical force or threat thereof, or in situations where the victim is unable to give consent [21,22,23]. The psychological trauma experienced as a result of sexual abuse may contribute to the development of CVD and its risk factors [24] through behavioural coping mechanisms. Psychosocial trauma, induced by sexual abuse, may initiate detrimental lifestyle behaviours of poor diet, decreased physical activity, smoking and excessive alcohol consumption. These, in turn, would lead to raised blood pressure (BP), obesity and predispose to diabetes and CVD [14,25,26,27].

Direct pathways via physiological mechanisms have also been postulated whereby acute and chronic stressors such as isolated or ongoing sexual abuse trigger a cascade of physiological responses that activate the cardiovascular, metabolic, neural, endocrine, autonomic and immune/inflammatory systems [23,24,28]. This in turn adversely affects BP, blood glucose and lipid levels, and promotes central obesity [14,27,28,29,30]; the likelihood of developing and worsening CVDs is increased.

Considering the high global prevalence of sexual assault (35% of women have experienced physical and/or sexual violence) [31], the potential severe psychological sequelae attributable to such trauma and the close association between psychosocial influences and CVDs, it is important to explore the relationship between sexual assault and the development of CVDs. Studies have shown a relationship between childhood sexual abuse (CSA) and the development of cardiovascular outcomes in adulthood [32]. However, adult sexual abuse (ASA) may activate different biological mechanisms compared with abuse experienced decades earlier during childhood. The impacts of psychosocial stressors are not uniform and the effects of one stressor cannot be generalised to another. Therefore, negative health outcomes associated with CSA may not be generalised to ASA. It is important to examine the effects of ASA separately from CSA while recognising that many individuals may be exposed to sexual abuse throughout their lifetime [32]. Similarly, experiences of ASA need to be evaluated independently of other traumatic experiences in adulthood such as physical or emotional abuse, divorce, death of a loved one, etc. This systematic narrative review seeks to describe the existing evidence regarding the association of ASA in women with the CVDs of heart attacks and strokes, and their cardiometabolic risk factors of hypertension, diabetes, dyslipidaemia and obesity.

## Methodology

### Sources of information and selection of eligible studies

The Preferred Reporting Items for Systematic Reviews and Meta-Analysis (PRISMA) framework was used for reporting this review [33]. A search of PubMed-Medline, Scopus and Web of Science was conducted of literature published till 1 October 2019. There were no limits on the earliest year of publication, and reference lists of articles identified were also examined for relevant studies. The literature was screened for any paper that examined the relation of ASA in women with the CVDs of heart attacks and strokes, and their cardiometabolic risk factors of hypertension, diabetes, dyslipidaemia and obesity. English language studies were obtained using key words such as ‘rape’, ‘sexual assault’, ‘sexual trauma’, ‘sexual abuse’, ‘sexual violence’ and ‘intimate partner violence’ (IPV) together with terms for individual CVDs as follows: 1) heart attacks: ‘heart disease’, ‘heart attack’, ‘cardiovascular disease’, ‘ischaemic heart disease’, ‘ischemic heart disease’, ‘coronary artery disease’, ‘coronary heart disease’ or ‘myocardial infarction’; 2) stroke: ‘stroke’ or ‘cerebrovascular accident’; 3) hypertension: ‘hypertension’, ‘high blood pressure’ or ‘raised blood pressure’; 4) lipids: ‘lipids’, ‘cholesterol’, ‘lipid’, ‘dyslipidaemia’ or ‘dyslipidemia’; 5) diabetes: ‘diabetes’, ‘diabetes mellitus’, ‘glucose’, ‘type 2 diabetes mellitus’ or ‘type 2 diabetes’; and 6) obesity: ‘obesity’, ‘obese’, ‘overweight’, ‘adiposity’ or ‘body mass index’.

The dearth of high-quality longitudinal data that examined ASA as the exposure of interest and diagnosed cardiovascular outcomes clinically resulted in the need for broad inclusion criteria; it was not possible to set rigorous criteria for the inclusion of studies. Therefore, studies were included irrespective of study design, i.e. longitudinal, cross-sectional, etc. However, included articles needed to have examined ASA per se independent of physical or psychological/emotional abuse, and independent of CSA, i.e. lifetime sexual abuse was excluded. This was to ensure that the impact of ASA was evaluated independently of other abuse forms because each abuse type is likely to differentially influence outcomes. Therefore, if ASA was assessed, even if other types of abuse were examined in the study, findings for ASA needed to be reported separately. This review did not apply any restrictions on the definition of the perpetrator, e.g., intimate partner or non-partner. Adult was defined as age ≥18 years of age.

### Data collection, extraction, assessment and synthesis

Two authors (NP, NA) conducted the literature search in duplicate and sequentially screened titles, abstracts and then full texts for inclusion. Any disagreements pertaining to the included papers were resolved through discussion or reviewed by the third author (APK). The reasons for excluding studies were also recorded. Studies were included irrespective of the data collection techniques for the selected outcomes i.e. clinically assessed, self-reported, or obtained from clinical records or national registries/databases, and irrespective of whether outcome definitions were included or not. Studies were excluded if a composite outcome measure was used for multiple conditions, e.g., hypertension, heart disease, diabetes, arthritis, AIDS, asthma, blindness, etc. Studies that included both men and women were included only if separate analyses were conducted by gender. If data from a study reporting the same exposure was published in more than one paper, the paper that presented the outcome data or contained more outcomes of interest was included.

The data extracted from the selected studies included variables relating to the study design and setting, sample size, participant characteristics, exposures examined, and outcomes evaluated. The exposure of ASA was self-reported in all studies but defined and assessed differently across studies. For example, some studies examined only IPV, i.e., sexual abuse per se or in combination with physical and/or emotional/psychological abuse while other studies specified sexual abuse in the military or assessed sexual abuse by any perpetrator. The timing of the sexual abuse varied from abuse in adulthood overall to specifically in older age or within the previous 12 months.

Outcomes included 1) physician diagnosed heart attack or stroke, 2) diagnosis by a health professional of hypertension, diabetes or dyslipidaemia with participants receiving treatment, and/or BP, glucose and lipid levels measured in the study, and 3) heights, weights, waist or hip circumferences measured in the study to determine obesity levels.

The quality of the included studies was assessed by adapting the criteria developed by Suglia and colleagues [32]. A point each was allocated for the following four criteria: 1) sample size >500 participants, 2) longitudinal follow-up for cardiovascular outcomes, 3) validated exposure measure e.g. Revised Conflict Tactics Scale (RCTS) and 4) objectively measured outcomes e.g. BP taken by the study team. A higher score was indicative of a higher quality study.

Ethical approval was not required for the analyses of these secondary data.

## Results

### Overview

**Figure 1:**
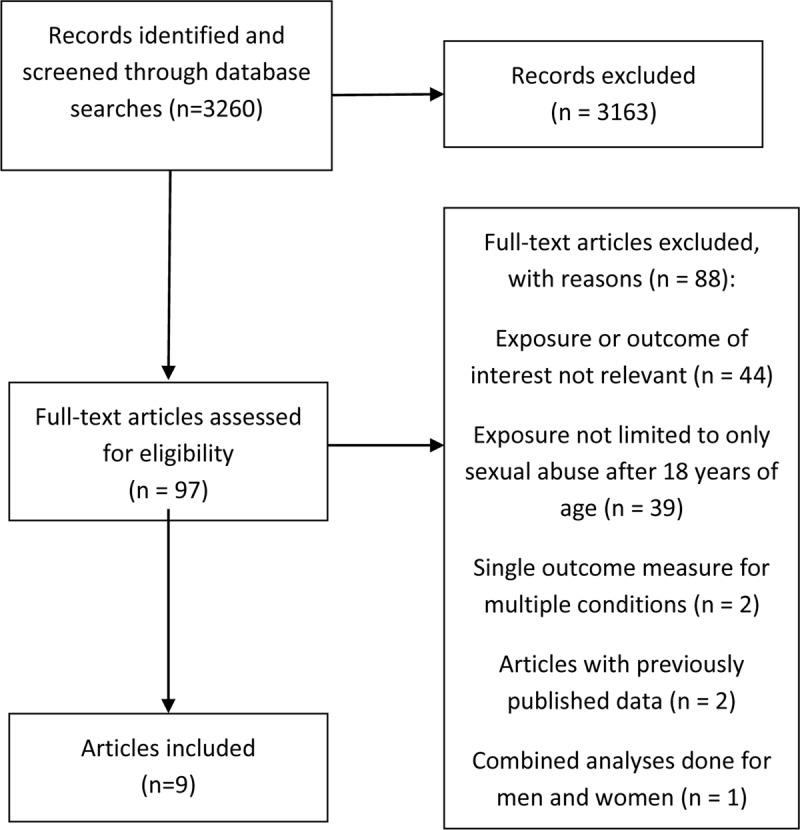
Flow diagram of the papers selected.

Of the 3260 records identified, 3163 were excluded because they did not cover the topic of this review, the exposures were not relevant (CSA, workplace harassment, maltreatment by partner, etc.) or the outcomes were not relevant (depression, cancer, pain, etc.), among other reasons. Ninety-seven full text articles were assessed for eligibility with nine articles eventually selected for inclusion in this review (Figure 1). A meta-analysis was not conducted due to heterogeneity across the selected studies. Definitions of exposures and outcomes differed across studies as did the sampled populations, study designs and data collection techniques for the outcomes.

The nine articles selected for the review of the relationship between ASA and the CVD outcomes of heart attack, stroke, hypertension, diabetes, dyslipidaemia and obesity are presented in Table 1, and comprised a total of 855 206 women. These included three longitudinal articles [34,35,36] and six cross-sectional studies [37,38,39,40,41,42]; all were conducted in the United States. It was not possible to infer causality from the cross-sectional studies. The quality of the included studies was generally sub-optimal with all but one study scoring 1–2 out of 4 points. Most studies comprised large sample sizes (n = 8), three studies were longitudinal in design, four used validated questions and only a single study objectively measured an outcome of interest.

**Table 1:** Overview of included papers.

1^st^ author, year; Study name	Participant recruitment and data collection technique	Number of women; Mean age/range; Length of follow-up in longitudinal studies	Exposure examined, and description of scales used, or questions asked	Outcomes examined	Overall study exposures and outcomes

**LONGITUDINAL: United States**					
Mason, 2012 [34] Nurses’ Health Study II (NHSII)	Registered female nurses; Mailed surveys	51 434; mean age: 52 years; 6-year follow-up	Sexual IPV in adulthood (age 18+); ‘Has your spouse/significant other ever forced you to have sexual activities?’	Self-reported hypertension	Exposures: physical, sexual and emotional IPV Outcomes: hypertension incidence
Mason, 2013 [35] NHSII	Registered female nurses; Mailed surveys	64 732; mean age 47 years; 6-year follow-up	Lifetime sexual IPV: ‘Has your spouse/significant other ever forced you to have sexual activities?’	Self-reported diabetes, BMI assessment was not described	Exposures: physical, sexual, and psychological IPV Outcomes: diabetes incidence
Mason, 2017 [36] Project EAT	Public middle and high school; Mailed questionnaires (baseline); Online or paper surveys (follow-up)	619; mean age: 19 years at baseline; ≥18 years at baseline; 5-year follow-up	Ever sexual IPV; sexual IPV questions derived from Sexual Experiences Survey	Self-reported height and weight to calculate BMI	Exposures: physical and sexual IPV; depressive symptoms Outcomes: weight-related health
**CROSS-SECTIONAL: United States**					
Frayne, 1999 [37] Veterans Administration (VA) Women’s Health Project	VA outpatient health services; Mailed survey	3,543; ≥18 years	Sexual assault while in the military; ‘Did you ever have an experience where someone used force or the threat of force to have sexual relations with you against your will while you were in the military?’	Self-reported treatment for a heart attack or stroke within the past year; angina or chest pain, other heart problems, hypertension, diabetes, obesity	Exposures: sexual assault while in the military Outcomes: a wide spectrum of physical symptoms and medical conditions
Bonomi, 2007 [38]	Health insurer enrolment files; Telephone survey	3,008; 18–64 years	Adult lifetime sexual IPV; questions from BRFSS survey	Collection of weight and height data to calculate BMI is not described	Exposures: sexual and physical IPV Outcomes: mental, social and physical health
Smith, 2010 [39] Epidemiologic STudy of HEalth Risk (ESTHER) Project	Community-based convenience sample of heterosexual and lesbian women	867; 35–64 years	Adult sexual abuse; ‘Since the age of 18, was there a time when someone forced you to have sexual activity that you really did not want?’	Clinical assessments of height and weight done to diagnose obesity	Exposures: sexual abuse and sexual orientation Outcomes: heart disease risk factors
Newton, 2011 [40]	Community-based convenience sample of 45–60-year-old post-menopausal women; questionnaire interviews and clinical measurements	68; mean age: 54.7 years	IPV: Sexual coercion experienced >1 year ago; assessed by the Revised Conflict Tactics Scale	Collection of weight and height data to calculate BMI is not described	Exposures: IPV: physical assault, sexual coercion, stalking, and psychological aggression Outcomes: biologic mediators of inflammation
Cichowski, 2017 [41]	Women treated by a VA primary care physician at any time since 1997; Clinical records obtained from American Veterans’ Health Administration Corporate Data Warehouse	516,950; mean age: 63.3 years (MST) mean age: 67.4 years (no MST)	MST; MST screening questionnaire	Average BMI calculated using average height and weight over time	Exposures: MST Outcomes: chronic pain conditions
Pandey, 2018 [42]	Veterans’ Health Administration database for 2014; Review of electronic medical records	213,985; ≥18 years Age range: 20–103 years	MST; MST screen or use of a specialty MST clinic in 2014	Anthropometric data from electronic medical records calculated BMI	Exposures: MST, PTSD Outcomes: obesity

BP: blood pressure; BMI: body mass index; IPV: intimate partner violence; MST: military sexual trauma; BRFSS: Behavioral Risk Factor Surveillance System; PTSD: post-traumatic stress disorder.

### Study populations, settings, participant recruitment and data collection

The included studies evaluated specific groups such as military veterans [37,41,42], nurses [34,35], enrolees with a specific health insurer [38], school attendees (at baseline) [36], heterosexual and lesbian women [39] and 45–60-year-old post-menopausal women who were divorced or separated from a stressful relationship [40] (Table 1). Participants in the included studies were selected from communities (convenience samples) [39,40], Veterans Administration (VA) clinics [37], schools [36] and databases or registries [34,35,38,42].

Across studies, different approaches were employed for data collection ranging from mail and telephone interviews with self-reported outcomes to onsite-administered questionnaires and objectively measured clinical assessments. The most frequently used data collection technique was telephonic interviews [38] followed by questionnaire interviews alone [40] or with clinical assessments [39]. Other data collection methods employed were postal surveys alone [34,35,37] or with review of electronic medical records [41,42]. A single included study used a combination of data collection methods; mailed questionnaires at baseline and online or paper surveys on follow-up [36].

### Assessment of outcomes and sexual abuse

Many studies included self-reported cardiovascular outcomes without confirmation of the diagnoses. Only a single included study performed any clinical assessments to determine the relevant outcomes, i.e., Smith et al. measured heights and weights to calculate obesity levels [39]; no study measured BP, glucose or cholesterol levels to determine hypertension, diabetes or dyslipidaemia, respectively (Table 2). Two studies gathered outcome data from clinical records i.e. electronic medical databases [41,42] and four relied on participants’ self-report to determine clinical outcomes [34,35,36,37]. Two articles did not provide details on outcome assessments i.e. they did not describe how weight and height data were collected [38,40].

**Table 2:** Distribution of included articles by type of abuse and outcomes (article references shown in the table).

Perpetrator of sexual abuse	Number of papers	Collection of outcome data	Heart attack (n = 1)	Stroke (n= 1)	Hypertension (n= 2)	Diabetes (n= 2)	Adiposity (n= 8)	Cholesterol (n= 0)

Any	One	Clinically assessed [39]					N/S [39]	
Intimate partner	Five	Self-report [34,35,36] Not described [38,40]			N/S [34]	N/S [35]	N/S [35,36,38,40]	
Military personnel	Three	Self-report [37] Medical records [41,42]	S [37]	N/S [37]	S [37]	N/S [37]	S [37,41,42]	

N/S: not significant, S: significant.

Sexual abuse by an intimate partner was examined in five articles with abuse evaluated using validated scales (the Sexual Experiences Survey and the sexual coercion section of the RCTS), questions from the Behavioral Risk Factor Surveillance System (BRFSS), and a single question in two studies (’Has your spouse/significant other ever forced you to have sexual activities?’). Three papers assessed sexual assault in the military [37,41,42] using the validated military sexual trauma (MST) screening questionnaire in two studies and an unvalidated single question in the third (’Did you ever have an experience where someone used force or the threat of force to have sexual relations with you against your will while you were in the military?’). A single paper studied sexual abuse since the age of 18 in heterosexual and lesbian women with the question ‘Since the age of 18, was there a time when someone forced you to have sexual activity that you really did not want?’ assessing abuse [39] (Table 2). Data on the timing of the ASA i.e. ‘how long ago had the abuse occurred’ was not collected in most studies. A single cross-sectional study asked about ASA that had occurred more than a year ago and examined the association with obesity. The longitudinal studies compared the current outcomes of self-reported incident hypertension [34], diabetes [35] and obesity [36] with ASA determined in surveys conducted six [34,35] and five years [36] earlier.

### Associations of ASA with CVDs and their cardiometabolic risk factors

A single article comprising 3,543 women veterans in the United States examined self-reported ‘heart attack’, angina or other cardiac problems within the previous year, as well as being treated for a stroke in the previous 12 months (Table 2). Being ‘treated for a heart attack within past 12 months’ (adjusted odds ratio [OR]: 2.3, 95% confidence interval [95% CI]: 1.4–4.0) was found to be significantly associated with sexual abuse while in the military after controlling for age, hypertension, diabetes and smoking status. However, sexual abuse while in the military was not significantly associated with ‘treated for a stroke within past 12 months’ (OR: 1.5, 95% CI: 0.8–2.9) after adjusting for age. This was a cross-sectional study which used a single-item unvalidated question for sexual abuse (‘Did you ever have an experience where someone used force or the threat of force to have sexual relations with you against your will while you were in the military?’) [37].

Two studies examined the association of self-reported hypertension with ASA; one study (n = 3,543) found the association to be significant (age-adjusted OR: 1.3, 95% CI: 1.1–1.6) [37] while the other (n = 51,434) did not (adjusted hazard ratio: 0.99, 95% CI: 0.91–1.07) [34]. The two studies, however, are not comparable. The study that described a significant association was cross-sectional, examined the association of sexual abuse while in the military with prevalent hypertension, and controlled for age only [37]. The other study, however, was longitudinal with a 6-year follow-up period, evaluated the association of IPV with incident hypertension in nurses, and controlled for age, body mass index (BMI) at age 18, and childhood physical and sexual abuse, among other variables [34]. Childhood abuse, apart from age, was found to be the most influential confounder of the association between ASA and hypertension.

The same two studies examined the association of self-reported diabetes with ASA with both reporting no significant relation (age-adjusted OR: 1.3, 95% CI: 0.9–1.7 [37]; adjusted hazard ratio: 1.02, 95% CI: 0.81–1.28) [35]. For obesity (n = 803,772), 3 vs. 5 articles reported significant vs. non-significant associations with ASA. The three studies that reported significant associations with obesity examined MST while four of the five studies that reported non-significant links evaluated IPV. None of the included studies examined dyslipidaemia.

Most studies adjusted for age, an important confounder of cardiovascular and cardiometabolic diseases, except for three studies [39,40,41]. The focus of two of the latter studies was not the outcomes of interest in this review but on biologic mediators of inflammation [40] and chronic pain conditions [41].

## Discussion

A systematic review of the literature was performed to determine the associations of ASA with CVDs of heart attack and stroke and their risk factors of hypertension, diabetes, dyslipidaemia and obesity. There is an absence of high-quality data that examined these associations. Only nine articles, all from the United States, were included in this review with a single study examining heart attack or stroke, two articles each on hypertension and diabetes, eight on adiposity and none on dyslipidaemia.

The available evidence, overall, is inconclusive with no clear associations demonstrated between ASA and CVDs; however, the direction of some associations indicates a potential relationship. The reasons for inconsistent findings in this review are numerous. These may include the use of self-reported outcomes with a lack of standardised definitions, differences in the types of exposures examined as well as differences in the populations examined and study methodologies.

Using self-reported information for a diagnosis of diabetes, hypertension, dyslipidaemia and obesity is unreliable and will most likely lead to under-reporting. Therefore, although studies on self-reported hypertension and diabetes reveal an absence of significant associations with ASA, these findings should not be regarded as conclusive. There is a need for objective assessments of CVD risk factors using standardised internationally recommended techniques for determining anthropometry, BP, glucose and cholesterol levels.

Interestingly, the three studies that reported significant associations with obesity all examined MST. However, one study reported a higher average BMI with MST vs no MST without examining further associations [41]. Another study adjusted for age only [37] while the third adjusted for demographics and multiple comorbidities [42]. Although little is known about the correlation of ASA with obesity, the few studies that have examined this association in women have highlighted the complexity of the relationship. Studies that have examined the association of ASA with obesity have evaluated different influences on the relationship which have altered the associations. For example, in the Nurses’ Health Study II, once childhood abuse was taken into consideration, IPV and BMI were no longer associated [35]. Bonomi et al who adjusted for age, income and any abuse in childhood also found no association between sexual IPV and BMI [38]. The ESTHER study reported a significant unadjusted association between ASA and obesity which disappeared once adjusted for sexual orientation [39]. In Project EAT, while there was no significant increase in BMI over five years between IPV exposure and no exposure, depressive mood was associated with an additional and significant increase in BMI in women with IPV exposure [36]. This highlights that multiple factors likely influence the relationship of ASA with obesity and may have contributed to the differential findings across studies. Therefore, there is a need to understand the behavioural and psychobiological responses to sexual abuse that influence weight and the development of obesity considering that the latter is a key contributor to CVDs. Further research is required to explore the influence of ASA on the development of obesity or weight change.

The different types of ASA in terms of perpetrator, severity, duration, etc. were not evaluated in the included studies and is beyond the scope of this review [44]. Each stressor at each level of intensity or severity, has a distinct ‘signature’ in the patterning of hormone levels [45], which will likely influence the impact of psychological stress on the development of CVD. Evaluating these differential psychosocial stress influences and the associations with CVDs may lead to a better understanding of the effect of ASA on long-term cardiovascular health.

Most of the included studies on ASA focused on IPV, which may or may not be experienced differently from abuse by a non-intimate partner. This prevents generalisability of any findings, as few studies included women who had ever experienced any form of ASA only. This is understandable for studies examining IPV which is frequently accompanied by psychological/emotional abuse.

The limitations of this review are that only English language papers were accessed and that a meta-analysis could not be conducted because of the heterogeneity across studies. The associations of sexual abuse in men with CVDs were not examined in this study and need to be included in future reviews. Because of limited resources and the likelihood of finding fewer studies in men on the topic under review, the focus was solely on women, the more vulnerable group. The strength of this study is that three databases were accessed for this review.

## Recommendations

Currently, no longitudinal studies of ASA in general populations have objectively investigated the development of CVD and its risk factors using clinical and biochemical measurements. This is likely because sexual abuse is difficult to research and that such studies require large samples of participants followed-up for a long duration. Tackling this complex topic requires the development of sensitive, rigorous, and longitudinal research methods to study ASA [46] and its impact on the development of CVDs. Such studies are required to elucidate any temporal relationship, particularly in the context of the high and rising worldwide burden of CVDs [47] and the substantial global prevalence of ASA [48].

A longitudinal study recruiting over a thousand rape-exposed and rape-unexposed women in each group is currently being undertaken in Durban, South Africa (Protocol ID: EC019-10/2013) [49]. Among the outcomes measures that are being clinically assessed are cardiometabolic variables of BP and anthropometry, and biochemically analyses of glycosylated haemoglobin (HbA1c) and lipid profiles, among other biomarkers. Although the follow-up duration of this study is only 24 months, it may nevertheless provide valuable insights. This will be among the first studies to provide comprehensive objectively collected cardiometabolic data in recently rape-exposed participants who are followed up regularly over a two year period.

The diverse forms of abuse and the wide range in severity, frequency and duration of each type of abuse makes it a challenge to link a disease outcome with exposure to ASA [24]. A better understanding of the nature of the sexual abuse in terms of age of occurrence, severity, victim-perpetrator relationship, chronic abuse, co-occurrence of multiple forms of violence, use of psychological coercion, etc. will enable a more comprehensive analysis of a causal link between ASA and CVD outcomes [23,46], if definitive associations do exist.

Further, exploring the cultural influences, the social context in which abuse occurs, the role of social support and coping mechanisms is important. These may help identify factors that exacerbate or moderate the development of psychological stress post-sexual abuse and the subsequent CVD risk. For example, social support i.e. supportive responses following disclosures of abuse may play a moderating role in reducing poor health outcomes and the risk of developing CVDs. A focus on such research may provide unique insights on the important contributors to women’s health and well-being as well as strategies to improve coping mechanisms in dealing with stress. In a broader context, this may also contribute to a greater understanding of the role of psychosocial stress in the development of CVDs.

If future research demonstrated strong evidence that women exposed to ASA developed CVDs, then effective health promotion and prevention strategies to improve cardiovascular health in women with a history of abuse will need to be developed [43]. Women exposed to sexual abuse will need to be identified as a potential high-risk population for developing CVDs and encouraged to undergo CVD screening. Nevertheless, identifying women with sexual abuse in the general population will need to be approached with extreme sensitivity. Additionally, strategies to address and reduce psychological stress are currently absent in the routine care of individuals with CVDs. Such strategies will need to be developed, in the context of CVDs, to address and reduce the psychological harm associated with ASA.

## Conclusions

Sexually abused women represent a large and particularly vulnerable population whose physical and mental health outcomes require greater attention [23]. In view of the high and increasing burden of CVDs, and the frequency of ASA, it is important to fully understand the relationship between the two. However, the current literature provides insufficient evidence to quantify the relationship, if any, between ASA and CVDs and their risk factors of diabetes, hypertension, dyslipidaemia and obesity. Among the factors contributing to these shortcomings are the use of mainly self-reported outcomes and a dearth of studies examining all facets of ASA. Therefore, despite a conceptual link between sexual abuse and CVD, substantial gaps of evidence exist, particularly for ASA per se at a population level. Longitudinal studies that fully explore all aspects of ASA and rigorously assess cardiovascular outcomes are needed to evaluate the influence of ASA on the development of CVDs.
